# Model and Experimental Study on Optical Fiber CT Based on Terfenol-D

**DOI:** 10.3390/s20082255

**Published:** 2020-04-16

**Authors:** Wang Li, Xia Kewen, Weng Ling

**Affiliations:** 1 School of Electronics and Information Engineering, Hebei University of Technology, Tianjin 300401, China; kwxia@hebut.edu.cn; 2 School of Electrical Engineering, Hebei University of Technology, Tianjin 300401, China; llweng@hebut.edu.cn

**Keywords:** current transformer, Terfenol-D, fiber Bragg grating, unbalanced M–Z interferometer

## Abstract

A nonlinear hysteresis model of magneto-mechanical-thermo coupling for Terfenol-D materials is presented according to Wiss ferromagnetic theory, thermodynamics relations and Jiles–Atherton model. Numerical calculation and experimental results show that the mode well reflects the magnetostrictive characteristics of Terfenol-D rod under the coupling of stress, temperature and magnetic field. A fiber Bragg grating current transformer based on Terfenol-D material is designed according to the strain sensing mechanism of fiber Bragg grating and the demodulation principle of unbalanced M–Z interferometer. The theoretical analysis and research on the working characteristics of the fiber current transformer under the influence of different prestressing force and bias current are carried out. The results are important for the design and application of the current transformer with the Terfenol-D material.

## 1. Introduction

High accuracy, safety, stability and reliability of high voltage and high current detection have always been the key to the measurement of power system, and the current transformer with optical fiber as sensing element has become a research hotspot in the measurement of power system with its advantages of high stability, reliability, antielectromagnetic interference, corrosion resistance, integration of sensing and transmission, compatibility with digital communication system, etc.

The optical current transformer based on the Faraday magneto-optical effect is studied in reference [1,2,3,4], which uses magneto-optical crystal/glass as a sensing element and optical fiber as the transmission link to realize 3000 A. The accuracy level of the current measurement within the range can reach 0.2. This kind of sensor has high sensitivity and simple structure, but its antielectromagnetic interference ability is poor due to the influence of the structure and sensing mechanism [5,6,7,8]. Although Faraday current transformer with an optical fiber ring structure can avoid electromagnetic interference, the birefringence of optical fiber limits its development and practical application [9,10,11]. In addition, the optical current transformer based on a magnetic fluid is a new type of current transformer, which has developed rapidly in recent years [12,13]. This transformer uses the magnetic controllability of magnetic fluid and SMS (Single mode-Multimode-Single mode)optical fiber structure to realize the measurement of current or magnetic field. It has the advantages of easy processing, strong controllability and low cost [14,15]. In addition, the optical current transformer based on the magnetostrictive material is an important research direction of optical current transformer because of its small size, light weight and large measurement range [16,17,18]. Fusiek et al. [19] studied the feasibility of the application of current/voltage transformer based on laminated piezoelectric composite in aircraft electrical system. The system uses arrayed waveguide grating for wavelength demodulation, which has the advantages of high stability and good dynamic performance. The combination of fiber grating and giant magnetostrictive material (Terfenol-D) is also an important direction of the development of optical current transformer in recent years [20,21,22,23]. The characteristic of this type of sensor is that it does not need polarization maintaining fiber and devices, and it is easy to be cascaded. It can form a fiber-optic sensor network. However, the wavelength demodulation technology that can reflect the wavelength change in real time and accurately is one of the key technologies that affect the measurement sensitivity and accuracy based on a giant magnetostrictive material and fiber grating current transformer. Literature [24,25] has carried out theoretical and experimental research on current sensors based on magnetostrictive materials and fiber gratings, and established a model that can reflect the magnetothermal coupling characteristics of Terfenol-D materials. The model can well describe the magnetostrictive characteristics of Terfenol-D materials at different temperatures, but the description of magnetostrictive characteristics of Terfenol-D materials under different prestress is not accurate, especially in the middle and low magnetic field region, the calculated results of the model are obviously different from the experimental results. In this paper, the more accurate magnetic thermal force coupling nonlinear hysteresis model of Terfenol-D material is established. Combined with the strain sensing mechanism of fiber Bragg grating and the demodulation principle of unbalanced M–Z interferometer, the fiber Bragg grating current transformer with Terfenol-D material as the core is designed. The main factors that affect the working characteristics of the current transformer, bias current and prestress, are analyzed and studied theoretically to obtain the best working condition.

## 2. Structure and Working Principle of Current Transformer Based on Terfenol-D

**Figure 1:**
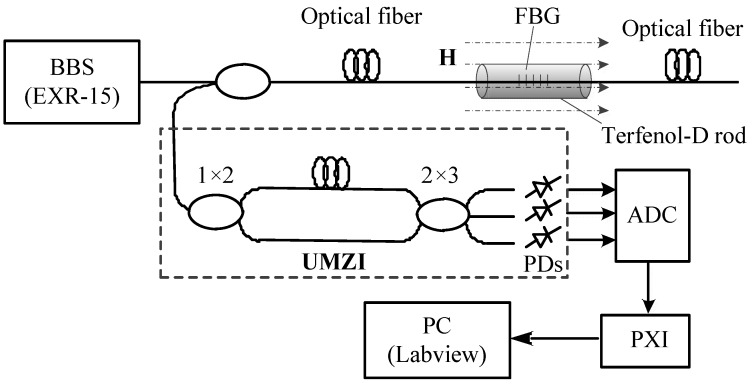
Composition of optical current transformer based on Terfenol-D.

The structure of the current transformer based on Terfenol-D is shown in Figure 1. Its working principle is as follows: The light from broadband light source (BBS) is incident to fiber Bragg grating (FBG), which is pasted on the Terfenol-D rod along axial direction through coupler. When the magnetic field or excitation coil current changes, dynamic axial magnetostrictive strain will be generated in Terfenol-D rod under the action of alternating magnetic field, and the fiber grating will produce a cooperative strain, the strain will cause the reflection central wavelength of the fiber grating to drift. The dynamic wavelength drift of FBG is demodulated by the unbalanced Mach Zehnder interferometer (UMZI).The structure of UMZI is shown in the dotted box in Figure 1, the principle of demodulation will be detailed in part II. The measurement of AC current or magnetic field can be realized by signal processing and display.

## 3. Model of Current Transformer

### 3.1. Multi Field Coupling Characteristics of Terfenol-D Materials

The relationship between temperature, stress, applied magnetic field and the strain of Terfenol-D material directly affects the accuracy of the current measurement. Therefore, it is necessary to establish the strain relation of Terfenol-D under the action of multi field coupling. According to Weiss ferromagnetic theory, the field that causes strain in Terfenol-D is the effective field inside the material, which is the sum of the external magnetic field and the molecular field, while the stress and temperature will cause the change of the effective magnetic field. Therefore, the effective field inside Terfenol-D can be expressed as:

(1) $$H_{e}=H+\alpha M+\frac{\partial \lambda }{\partial M}+\frac{2\beta t\sigma M}{\mu_{0}M_{S}^{2}}$$

In the Equation (1), the first item H is the external magnetic field. The second item αM is the magnetic field produced by the interaction between the magnetic domains of the material, the third item is the magnetic field generated by the rotation of the domain caused by stress to the plane perpendicular to the axial direction [26], and the fourth item is the coupling magnetic field generated by the interaction of prestress σ and temperature t [27]. Among them, α is the domain wall interaction coefficient, λ is magnetostriction and β is temperature independent magnetic-force-thermal coupling constant, $\mu_{0}$ is vacuum permeability, M is magnetization and $M_{S}$ is saturation magnetization at 0 °C.

The empirical model of the relationship between λ and M is given as [27]:

(2) $$\lambda =\left[1-tanh(2\sigma /\sigma_{s})/2\right]\frac{\lambda_{s}}{M_{s}^{2}}M^{2}+2\beta t\frac{M^{2}}{M_{s}^{2}}$$

Here, $\sigma_{s}$ represents the axial prestress value, which is determined by the strain–stress curves. $\lambda_{s}$ represents saturation magnetostriction, which can be measured by experiment. Therefore, the key to solve the magnetostriction of Terfenol-D by Equation (2) is to determine the magnetization of Terfenol-D under different stress, temperature and magnetic field.

Based on the Weiss molecular field theory and micromagnetism theory, Jiles and Atherton put forward the theoretical ferromagnetism model of J-A [26,28].

(3) $$M_{\mathrm{an}}=M_{S}\left[coth\left(\varpi H_{e}\right)-\frac{1}{\varpi H_{e}}\right]$$

(4) $$M_{rev}=\tilde{c}\left(M_{an}+M_{irr}\right)$$

(5) $$\frac{\partial M}{\partial H}=\frac{M_{an}-M_{irr}}{\delta k-\alpha \left(M_{an}-M_{irr}\right)}$$

(6) $$M=M_{irr}+M_{rev}$$

Here, $M_{an}$ is no hysteresis magnetization, $M_{irr}$ is irreversible magnetization, $M_{rev}$ is reversible magnetization and k and $\tilde{c}$ are irreversible loss coefficient and reversible coefficient respectively. The saturation magnetization $M_{S}$ is temperature-related, which can be written as $M_{S}\left(t\right)=M_{s}{\left[\left(t_{c}-t\right)/t_{c}\right]}^{0.5}$, where $t_{c}$ is Curie temperature 380 °C and $M_{S}\left(t\right)$ is the value of saturation magnetization at the actual temperature. $\varpi =\mu_{0}M_{S}\left(t\right)/Nk_{B}\left(t+273\right)$, where N is domain density, $k_{B}$ is Boltzmann constant. Here, δ is used to ensure that the theoretical calculation is consistent with the physical properties of Terfenol-D.

(7) $$\delta =\{\begin{array}{cc} 1 & dH/dt>0\\ -1 & dH/dt<0 \end{array}$$

The change of magnetization M can be solved by the Newton iterative method, so that the magnetization can be calculated. The relationship between the strain of Terfenol-D material and the applied current or magnetic field can be obtained by (1)–(6).

### 3.2. Principle of Dynamic Strain Demodulation of FBG

The FBG adhered along the axis of Terfenol-D rod will produce the same strain as the rod under the action of external magnetic field. According to the sensing mechanism of FBG, the strain will lead to the shift of resonant wavelength of FBG. The relationship between the wavelength shift $\Delta \lambda_{B}$ and the axial strain of FBG ε is [29]:

(8) $$\Delta \lambda_{B}/\lambda_{B}=K_{\varepsilon }\cdot \varepsilon$$

Here $K_{\varepsilon }=0.784$ is strain sensitivity coefficient of FBG. Under the condition that Terfenol-D rod, adhesive layer and FBG are closely combined and deformation is coordinated, the magnetostriction is equal to the axial strain of FBG, then relation (8) can be rewritten as:

(9) $$\Delta \lambda_{B}/\lambda_{B}=K_{\varepsilon }\cdot \lambda$$

Terfenol-D rod will produce dynamic strain under the action of time-varying magnetic field. Due to the coordination of FBG and Terfenol-D rod’s strain the real-time change of FBG central wavelength will reflect the change of time-varying magnetic field. Therefore, the demodulation system of FBG dynamic wavelength must have the characteristics of high resolution and good dynamic performance. In this paper, unbalanced Mach Zehnder interferometer was used as FBG dynamic wavelength demodulation system, the structure of the instrument is shown in the dotted box in Figure 1. The system consists of two sections of optical fiber (length difference is ΔL), input coupler (1×2) and output coupler (2×3). After the output signal of FBG is input into the unbalanced M–Z interferometer by the coupler, the change of its central wavelength will be converted into phase difference output, which can be expressed as [30]:

(10) $$\Delta \varphi =\arctan\left[\frac{\left(\mu_{2}-\mu_{3}\right)V_{1}/\alpha_{1}+\left(\mu_{3}-\mu_{1}\right)V_{2}/\alpha_{2}+\left(\mu_{1}-\mu_{2}\right)V_{3}/\alpha_{3}}{\left(\gamma_{2}-\gamma_{3}\right)V_{1}/\alpha_{1}+\left(\gamma_{3}-\gamma_{1}\right)V_{2}/\alpha_{2}+\left(\gamma_{1}-\gamma_{2}\right)V_{3}/\alpha_{3}}\right]$$

The phase difference generated by two optical fibers in the unbalanced M–Z interferometer is:

(11) $$\Delta \varphi =\frac{2n\pi \Delta L}{\lambda_{B}}$$

where μ, α and γ are dimensionless parameters, which can be determined by the method described in reference [30], $V_{1}$, $V_{2}$ and $V_{3}$ are voltages of photodetector connected at output end of coupler 2×3. According to the above analysis, we can get Δφ according to $V_{1}$, $V_{2}$, $V_{3}$, then the wavelength change can be calculated by Equation (11), so as to realize wavelength demodulation.

The mathematical model of current sensing system based on Terfenol-D material, FBG and unbalanced M–Z interferometer can be obtained by combining Equations (1)–(6) and (9).

(12) $$\Delta \varphi =\frac{2n\pi \Delta L}{\lambda_{B}+K_{\varepsilon }\left\{\left[1-tanh(2\sigma /\sigma_{s})/2\right]\frac{\lambda_{s}}{M_{s}^{2}}M^{2}+2\beta t\frac{M^{2}}{M_{s}^{2}}\right\}\cdot \lambda_{B}}$$

The relationship between M and H can be obtained by the relational expression (1)–(6).

From the above analysis, it can be seen that the output of the designed optical fiber current sensor depends on n, ΔL, $\lambda_{B}$, $K_{\varepsilon }$ and the relationship between M and H. When the structure of the optical fiber grating and the unbalanced M–Z interferometer is fixed, the working characteristics of the transformer depend on the magnetostrictive strain characteristics of the Terfenol-D rod under the influence of different stress and temperature. Therefore, the study of the magnetostrictive properties of Terfenol-D rod under different stress conditions will play a guiding role in the practical application of the sensor.

## 4. Experimental Results and Analysis

**Figure 2:**
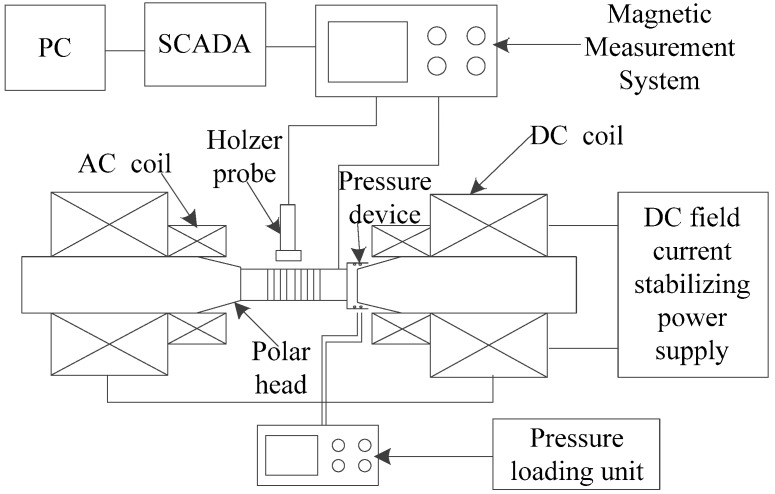
Testing system of multi field coupling characteristics of Terfenol-D.

In order to verify the feasibility of the current measurement scheme and the correctness of the established model, the magnetostrictive characteristics of Terfenol-D and the fiber Bragg grating AC current transformer under different stress and temperature are studied. The experimental device (As shown in Figure 2) is mainly composed of four parts: the magnetic material characteristic test system (multiparameter magnetic measurement system), gas pressure loading system, intelligent temperature control system and fiber grating sensing system. The Terfenol-D rod with a length of 18 mm and diameter of 8.4 mm was used in the experiment. In the magnetic material characteristic test system, two groups of coils, DC and AC, are respectively wound inside the two magnetic heads. When the system is loaded with current, a uniform axial magnetic field with a diameter of 100 mm can be generated between the two magnetic heads. The system can be loaded with a maximum of 50 A current, and a maximum of 700 kA/m uniform magnetic field can be generated between the two magnetic heads with a spacing of 100 mm. The measurement accuracy can reach 0.001 T. The gas pressure loading system can symmetrically generate 20 MPa uniform pressure between the left and right poles, and the loading accuracy can reach 0.01 MPa. The intelligent temperature control system composed of electric heating tube, temperature sensor and temperature controller can provide the temperature environment from room temperature to 100 °C, and the temperature control accuracy is ±1 °C. The temperature of electric heating tube can be continuously adjustable through the sensor and temperature controller. The system uses the corresponding software to control the measurement process and carry out data acquisition, such as the magnetic field is measured and data acquisition through Hall probe and Gauss meter, the strain of Terfenol-D rod is displayed by digital bridge through strain gauge measurement, etc.

At the same time, according to the model established in the second part, the output results of each part of the current transformer under a different temperature and stress were simulated. The calculation process was as follows:

(1) Determine the model parameters, set the prestress and temperature and set the simulation time $t_{s}$ and sampling period $T_{S}$, then the maximum simulation times is $m=t_{s}/T_{S}$, At first we made k=0;
(2) Solve Equations (3)–(6) using the Euler method and Newton iteration method, then the magnetization M(k) at time $t_{k}$ could be obtained;
(3) Solving magnetostriction λ(k) at time $t_{k}$ according to the relationship between λ and M, i.e., Equation (5);
(4) Solving FBG center wavelength variation $\Delta \lambda_{B}\left(k\right)$ at time $t_{k}$ according to Equation (9);
(5) Solving the phase difference Δφ(k) of an unbalanced M–Z interferometer at time $t_{k}$ according to Equation (12);
(6) k=k+1, if k<m, return to step (2), otherwise end.

The parameters of Terfenol-D used in the calculation were [31] $M_{s}=1.65\times 10^{5}$ A/m , $\lambda_{s}=1000\times 10^{-6}$, k=7000 A/m, $\tilde{c}=0.2$, α=0.0065, $\beta =5\times 10^{-6}$, $\mu_{0}=4\pi \times 10^{-7}$ H/m , $N=6.23\times 10^{23}$ and $k_{B}=1.38\times 10^{-23}$. The relevant parameters were optimized by a search algorithm [32] according to the experimental results.

The parameters of FBG and the unbalanced M–Z interferometer were [29,30] $K_{\varepsilon }=0.784$, $\lambda_{B}=1550$ nm, nΔL=3 mm and the fiber length was 1.5 km.

### 4.1. Experimental Results and Analysis of Multi Field Coupling Characteristics of the Terfenol-D Material

**Figure 3:**
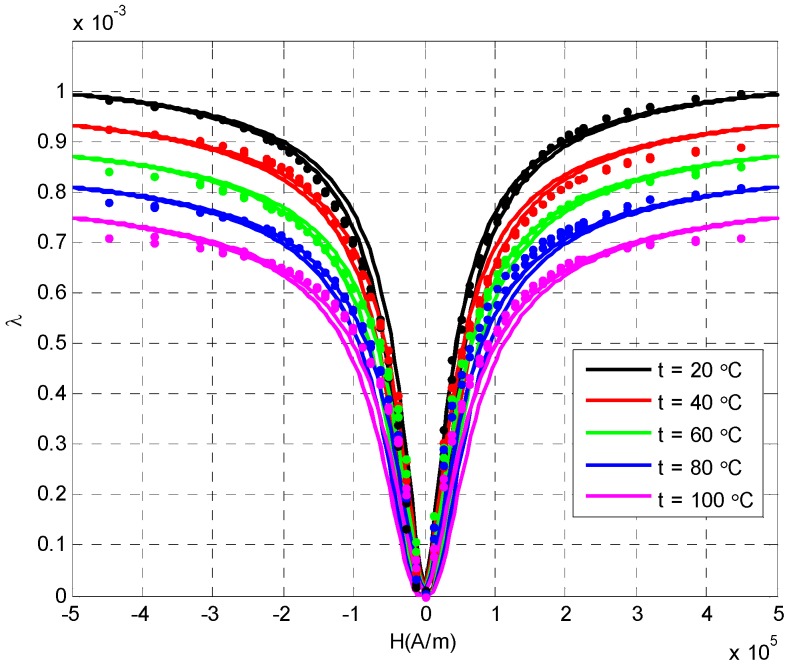
The magnetostriction dependences on applied magnetic fields of the Terfenol-D under different temperature (σ=0).

In order to test the dynamic response characteristics of current transformer under the influence of different temperatures, we firstly set the temperature to heat the rod. When the temperature of the Terfenol-D rod reached the set value, measurements were started: the current was applied in the excitation coil, the current increased from 0 to 35 A, and the corresponding magnetic field increased from 0 to 500 kA/m at the same time, measurements of the magnetostrictive strain of Terfenol-D rod were made, the current direction was changed and the above process was repeated. Figure 3 shows the strain curve of the Terfenol-D bar under the condition of 20 °C, 40 °C,60 °C, 80 °C and 100 °C without prestressing. The continuous curve was the numerical calculation result of the model, and the curve composed of discrete points was the actual test result. It can be seen from the figure that the calculation results of the dynamic hysteresis nonlinear model of the Terfenol-D material considering the multi field coupling characteristics were in good agreement with the measured results. The results show that the dynamic model could reflect the hysteresis and nonlinear characteristics of Terfenol-D material well under the condition of considering the influence of temperature on the main parameters of Terfenol-D material. At the same time, it could be seen from Figure 3 that temperature had little effect on magnetostrictive strain in low field area (H < 140 kA/M), and had great effect on magnetostrictive strain in high field area, especially the effect of saturated magnetostrictive strain was very obvious, and the saturated magnetostrictive strain would decrease with the increase of temperature, which will lead to the corresponding decrease of the magnetic field strain linear area. The current transformer with Terfenol-D material as the sensing element mainly used the linear region of magnetic field and strain, which is the low field region in the figure. Therefore, in the practical application of current sensor, when the change of ambient temperature was not too large, the influence of temperature on the sensing characteristics could be ignored. At the same time, in order to obtain a larger measuring range, we tried to choose the room temperature (20 °C) for measurement.

**Figure 4:**
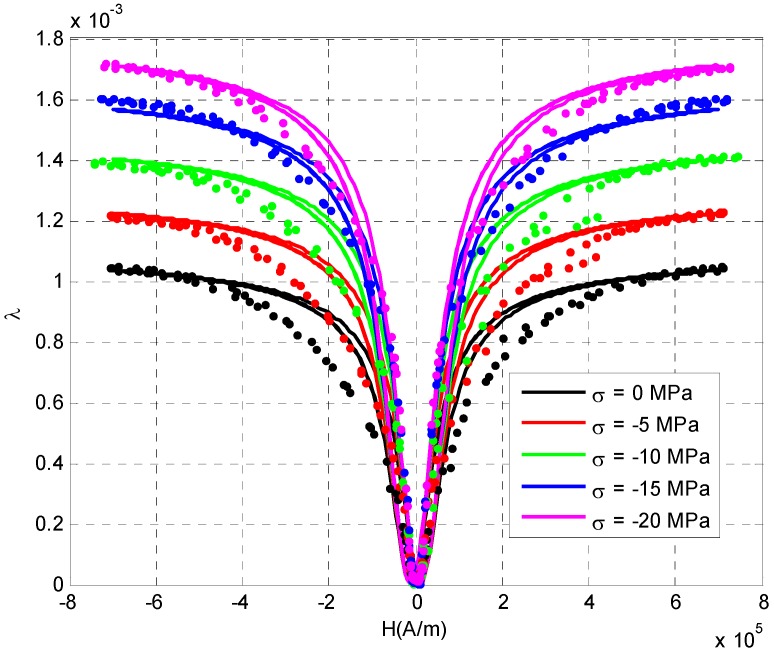
Room temperature magnetostriction corresponding to external magnetic fields of the Terfenol-D under different prestresses.

At room temperature (t = 20 °C) and under the influence of different stress, the test process of Terfenol-D magnetic field strain characteristics was similar to that under different temperature. Firstly, a certain pre pressure was applied to the Terfenol-D rod through the pressure loading device, and the measurement was started after the pressure was stable. Figure 4 shows the relation curve between the magnetic field and strain of Terfenol-D under the conditions of 0 MPa, −5 MPa, −10 MPa, −15 MPa and −20 MPa respectively. The continuous curve was the numerical calculation result and the discrete point was the actual measurement result. It can be seen from the figure that the prestressing force not only affected the saturated magnetostrictive strain of Terfenol-D, but also affected the magnetic field strain linear range. With the increase of prestress, the strain and linear range of magnetostriction increased obviously. This is due to the fact that the prestressing force caused the internal domain of the Terfenol-D material to rotate in a plane perpendicular to the axis, while the external magnetic field caused the domain to rotate in an axial direction. The larger the prestressing force is, the larger the magnetic field to overcome the prestressing force is. The slope of the curve between magnetic field and strain in the low field area decreased slightly, but the linear area increased obviously, and the saturated magnetostrictive strain in high field also increased with the increase of prestress. Therefore, when Terfenol-D was used to measure the current or magnetic field, the measurement range of the sensor could be increased by prestressing, at the cost of slightly decreasing the sensitivity of the sensor. In practical application, the prestress could be reasonably selected according to the needs of measurement.

In addition, it can be seen from Figure 3 and Figure 4 that the relationship between the magnetostrictive strain and external magnetic field was an even function. Therefore, in the process of actual current or magnetic field measurement, it is necessary to select an appropriate working point through DC bias to make the current sensor work in the linear area, ensure the measurement accuracy and avoid "double frequency" or nonlinear distortion of the measurement signal.

### 4.2. Experimental Results and Analysis of Optical Current Transformer Based on the Terfenol-D Material

According to the above theoretical analysis and experimental verification, it can be seen that the core of optical current transformer Terfenol-D material has obvious saturation and non-linear characteristics, and its magnetostrictive strain is only related to the magnetic field, and independent to the direction of the magnetic field, which will lead to the obvious phenomenon of "frequency doubling" in the process of measuring the AC signal. Therefore, how to adjust the measuring range by applying prestress and how to avoid nonlinear distortion by setting a static working point reasonably is the key to realize the accurate measurement of current transformer. In this section, we would focus on the influence of different prestress and bias current on the output characteristics of current transformer to guide its application in engineering practice.

**Figure 5:**
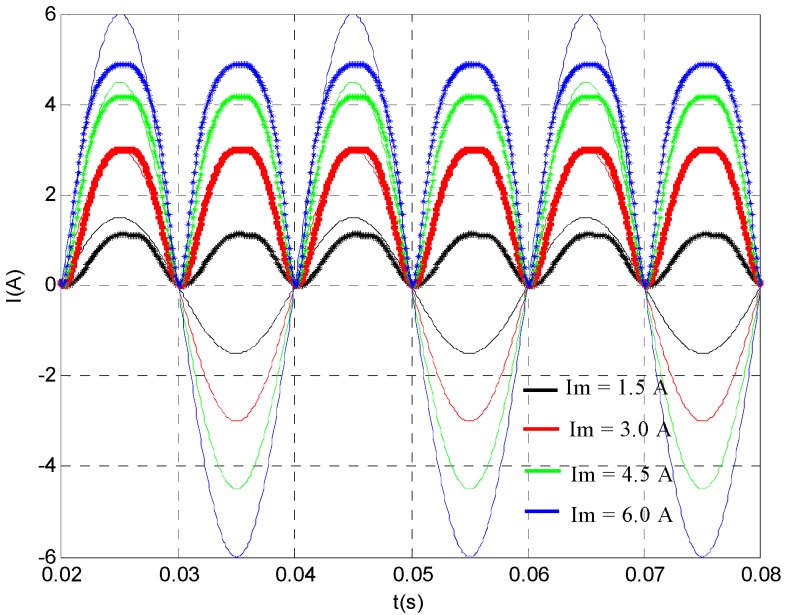
The relationship between the input and output of the AC current transducer with different input current amplitudes (*σ* = 0 MPa and *I*_b_ = 0 A).

When the bias current and prestress were both zero, we gradually increased the amplitude of sinusoidal alternating current with frequency of 50 Hz, the curve of comparison between the output and input of AC current transformer is shown in the Figure 5. The solid line is the current to be measured and the dotted line is the measurement result. It can be seen from the figure that no matter what the measured current was, the output had an obvious "double frequency" phenomenon. Obviously, this was caused by the even function relationship between the magnetostrictive strain of the Terfenol-D material and the current (magnetic field). Therefore, in order to avoid the phenomenon of "double frequency" in the measurement of the AC signal, a certain bias current (magnetic field) must be applied. When the amplitude of the current to be measured was small and the corresponding magnetic field was low (less than 3 kA/M), the output of the current transformer had obvious distortion (the output was obviously smaller than the input). This is because the Terfenol-D material was in the initial magnetization stage, the magnetostriction was small, and there was obvious non linearity between the input and output. With the increase of the current to be measured, the Terfenol-D material worked in the linear region, and the amplitude of the output current was almost the same as that of the input. However, with the further increase of the current amplitude to be measured, the Terfenol-D material entered into the saturation region, and the output current amplitude was obviously smaller than the current to be measured, and the saturation distortion of the transformer appeared.

**Figure 6:**
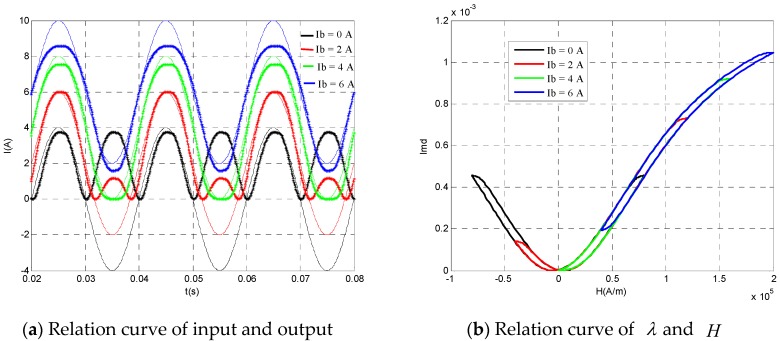
The relationship between the input and output of AC current transducer under different bias current (*σ* = −10 MPa and *I*_m_ = 4 A).

When the prestress σ = −10 MPa and the current amplitude to be measured $I_{m}$ = 4 A, Figure 6 shows the comparison between the output of the transformer and the current to be measured and the bias current was different. Obviously, without bias current ($I_{b}$ = 0 A), The output of the transformer had obvious "frequency doubling" phenomenon. At this time, the Terfenol-D magnetic field strain curve is the black hysteresis curve shown in Figure 6b, which had obvious symmetry. With the increase of bias current, the negative half axis part of the Terfenol-D hysteresis curve decreased, the positive half axis part increased and the frequency doubling phenomenon was weakened. When the bias current increased to 4 A, Terfenol-D works in the linear area, and the frequency doubling phenomenon disappeared, the output was almost the same as the input, at this time the transformer had the best working performance. As the bias current continued to increase, although there was no frequency doubling phenomenon, the maximum value of Terfenol-D hysteresis curve entered into the saturation region, resulting in obvious distortion in the upper half part of the measurement results. Therefore, it is very important to select the appropriate bias current for the current transformer with Terfenol-D as the core material.

**Figure 7:**
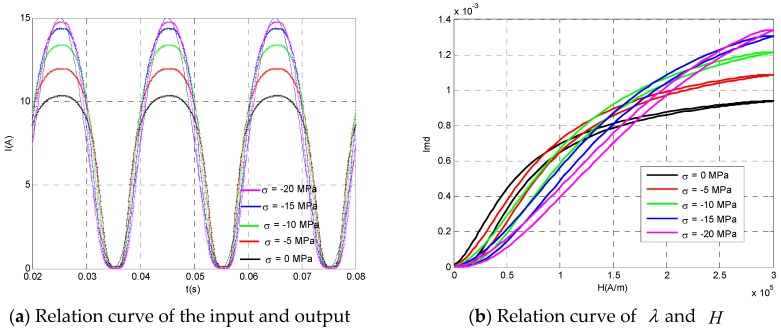
The relationship between input and output of the AC current transducer under different prestresses (*I*_b_ = 7.5 A and *I*_m_ = 7.5 A).

Figure 7 shows the relationship between the output of AC current transformer and the current to be measured when the bias current $I_{b}$ = 7.5 A, the current to be measured $I_{m}$ = 7.5 A, and the prestressing force σ = 0 MPa, −5 MPa, −10 MPa, −15 MPa, and −20 MPa, respectively. When the prestressing force was small, the output of the transformer had obvious saturation distortion. This is because the change range of the current to be measured exceeded the linear region of the Terfenol-D material, so there was an obvious phenomenon of “top clipping” in the upper half of the output signal. With the increase of prestress, the linear region of Terfenol-D material increased, and the upper half of the output signal gradually approached the current to be measured. When the prestress increased to 20 MPa, the output signal was almost the same as the current to be measured well, which indicates that the transformer was in the best working condition at this time. However, it can be seen from Figure 7b that although the linear region of Terfenol-D material would increase with the increase of prestress, the slope of the magnetic field strain curve would decrease with the increase of prestress, which indicates that the application of prestress would increase the measuring range of the device and reduce the sensitivity of the device. Therefore, in the engineering application, the appropriate prestress could be applied according to the measurement needs to make the device work in the best state.

In summary, both bias current and prestress would affect the working characteristics of the transformer. Therefore, in the process of design and application of AC current sensor, the influence of bias current and prestressing force on the working characteristics of the sensor should be considered comprehensively, and the values of bias current and prestressing force should be selected reasonably to make the sensor work in the best condition. The current transformer based on FBG has the characteristics of wavelength coding and temperature insensitivity, so it can be applied in hard environments and it can be combined with other grating sensors to form a sensor network to achieve a multipoint and multiparameter measurement. 

## 5. Conclusions

In order to study the working characteristics of the optical fiber current transformer based on the Terfenol-D material, an improved magnetic-thermal-force coupling nonlinear hysteresis model of Terfenol-D material was established according to the principle of Weiss, the relationship between ferromagnetic theory and thermodynamics, and the basis of Jiles Aiherton magnetization model. The numerical method was used to calculate the improved model, and the comparison with the experimental results shows that the model could more accurately reflect the magnetostrictive characteristics of Terfenol-D rod under the coupling of stress, temperature and magnetic field, especially the magnetostrictive properties of Terfenol-D materials in the middle and low magnetic field region under different prestresses. In addition, a fiber Bragg grating current transformer with the Terfenol-D material was designed based on the strain sensing mechanism of fiber Bragg grating and the demodulation principle of unbalanced M–Z interferometer. The theoretical analysis of the working characteristics of the fiber Bragg current transformer under the influence of different prestressing forces and bias currents was carried out. The results show that the transformer could measure AC current with different amplitudes by changing the prestressing force and bias current. The research could provide theoretical guidance for the optical fiber current transformer with Terfenol-D.
